# Novel biomarkers of fetal and neonatal environmental exposure, effect and susceptibility

**DOI:** 10.1038/s41390-025-03816-5

**Published:** 2025-02-12

**Authors:** Eric S. Peeples, Eleanor J. Molloy, Cynthia F. Bearer

**Affiliations:** 1 Pediatrics, University of Nebraska Medical Center, Omaha, NE USA; 2 Neonatology, Children’s Nebraska, Omaha, NE USA; 3 Child Health Research Institute, Omaha, NE USA; 4 Paediatrics, Trinity College Dublin, Trinity Research in Childhood Centre (TRiCC), Dublin, Ireland; 5 Trinity Translational Medicine Institute (TTMI), Dublin, Ireland; 6 Neonatology, Coombe Women’s and Infants University Hospital, Dublin, Ireland; 7 Neonatology, CHI at Crumlin, Dublin, Ireland; 8 Children’s Hospital Ireland (CHI) at Tallaght, Dublin, Ireland; 9 UH Rainbow Babies & Children’s Hospital, Cleveland, OH USA; 10 Case Western Reserve University School of Medicine, Cleveland, OH USA

## Abstract

**Abstract:**

Rapid advancements in science and technology have allowed medical providers to treat wider ranges of diseases with safer and more effective therapies than ever before. One of the areas of health that has been consistently understudied, however, is one that affects us all: environmental health or the effects that the chemicals we are exposed to every day have on our acute and chronic health. This effect can be exacerbated during and shortly after pregnancy, as an individual exposure is often shared by both the mother and the fetus/neonate. The diagnosis and monitoring of chemical exposure can be quite challenging, and improving our understanding of the effects of exposure will therefore require effective use of an expanding set of biomarker tests and biological matrices. This review covers the background and history of neonatal biomarkers of exposure, effect, and susceptibility, focusing on the potential uses for the non-invasive matrix of exhaled breath for the detection and monitoring of chemical exposures.

**Impact:**

- Provides a brief overview of Food and Drug Administration and National Institutes of Health Joint Leadership Council BEST (Biomarkers, EndpointS, and other Tools) Resource.
- Summarizes new and potential biomarkers for fetal exposure.
- Collates studies using breath as a matrix for environmental exposures.

## Introduction

Environmental health encompasses a broad range of disciplines, including ecology, geology, meteorology, biology, chemistry, medicine, engineering, epidemiology, biostatistics, and physics to study the complex impacts of the environment on human health. Environmental factors are increasingly universally recognized as important in human health, driven in large part by considerable public health efforts to raise general awareness around issues ranging from the dangers of tobacco smoke to health effects of global climate change. Although deforestation and soil erosion have caused climate crises and devastating effects on humans for millennia, the study of the environment became more prominent in the Age of Enlightenment of the 17th and 18th centuries with prominent figures such as Benjamin Franklin, who campaigned against water pollution, and George Baker who discovered the source of the Devonshire colic (lead in cider). Despite the long history of environmental exposure, however, many questions remain unanswered. More rapid progress in the field has been hampered by multiple factors, but one of the most persistent is the lack of reliable biomarkers to detect relevant environmental exposures.

A biomarker is defined by the Food and Drug Administration (FDA) and National Institutes of Health (NIH) Joint Leadership Council BEST (Biomarkers, EndpointS, and other Tools) Resource as a “characteristic that is measured as an indicator of normal biological processes, pathogenic processes, or biological responses to an exposure or intervention, including therapeutic interventions. Biomarkers may include molecular, histologic, radiographic, or physiologic characteristics. A biomarker is not a measure of how an individual feels, functions, or survives.”^1^ The measurement and monitoring of biomarkers of chemical exposure in human matrices such as urine, blood, or hair is often referred to as human biomonitoring (HBM). The Horizon2020 European Human Biomonitoring project HBM4EU is developing a European wide approach to standardized measurements.^2^ HBM becomes more complex when evaluating maternal/fetal exposures and specifically when attempting to estimate fetal and early neonatal exposures from neonatal matrices.

This review will explore the history and the standards of biomarker development and focus on two areas: matrices with which to measure fetal/neonatal biomarkers of environmental exposure/effect, and the specific use of breath as a matrix in maternal/perinatal HBM. For common fetal/neonatal chemical exposures, see the Biomonitoring Data Tables from the Centers for Disease Control and Prevention.^3^

## History

The use of biomarkers in environmental health sciences dates back to the 1960s and 70 s with Dr. Herbert Needleman’s pioneering work using lead in teeth as biomarker for lead neurotoxicity in children^4^ and the work of Dr. Harada measuring placental and umbilical cord concentrations of mercury of the infants with Minamata disease.^5^ The concept that a proxy measure could be used to estimate exposure was adopted by the pediatric environmental health community and incorporated into the terminology for biomarker research developed by Columbia University’s Drs. Frederica Perera and I. Bernard Weinstein. They proposed the term molecular epidemiology for the use of biomarkers to link human environmental exposures to cancer and suggested that exposure assessment should turn its focus from population-based estimates of dose to estimates of biologically effective dose—defined as the amount of a chemical interacting with critical cellular targets—within individuals.^6^

**Fig. 1:**
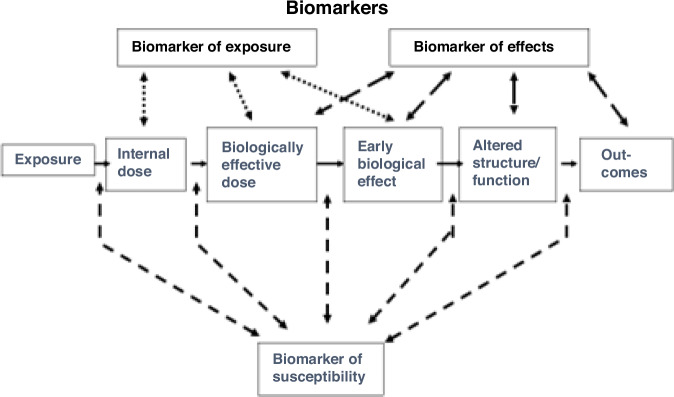
Representation of the continuum of biological events model, demonstrating the types of biomarkers that could be used to delineate each event within the continuum (from exposure to clinical disease outcomes).

Public concern over the health effects of environmental pollutants spurred the Board on Environmental Studies and Toxicology of the National Research Council’s Commission on Life Sciences to undertake a major investigation into the use of biomarkers in health research beginning in 1987. As part of this work, the Committee on Biological Markers was formed at the request of the Office of Health Research of the U.S. Environmental Protection Agency, the National Institute of Environmental Health Sciences, and the Agency for Toxic Substances and Disease Registry to clarify the concepts and definitions of biological markers. In one of the committee’s first publications, they set out a classification system of biological markers into markers of exposure, effect, and susceptibility.^7^ It also introduced the concept of a “continuum of biological events,” proposing that biomarkers could be used to delineate each event within the continuum, from exposure, to internalized dose, to biologically effective dose, to altered molecular structure, and finally to clinical disease (Fig. 1).

**Fig. 2:**
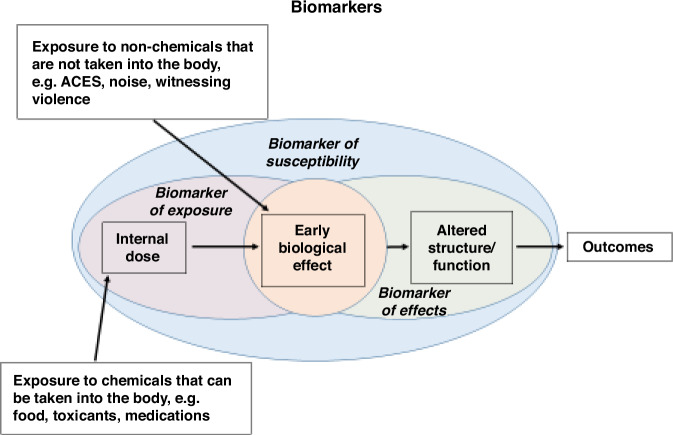
Modification of the continuum model of health to include chemical and non-chemical (such as noise, adverse childhood experiences (ACES), etc.) exposures that could lead to biologic change.

As a forerunner of the National Children’s Study, the National Research Council and Institute of Medicine, with funding from the Department of Health and Human Services, created the Committee on Evaluation of Children’s Health which further expanded on the continuum of biological events. This committee was charged with defining children’s health and investigating ways to assess and improve child health, including the role of biomarkers.^8^ This examination led to the inclusion of health influences beyond chemical and non-chemical (such as noise, adverse childhood experiences, etc) exposures that could lead to biologic changes (Fig. 2).

**Fig. 3:**
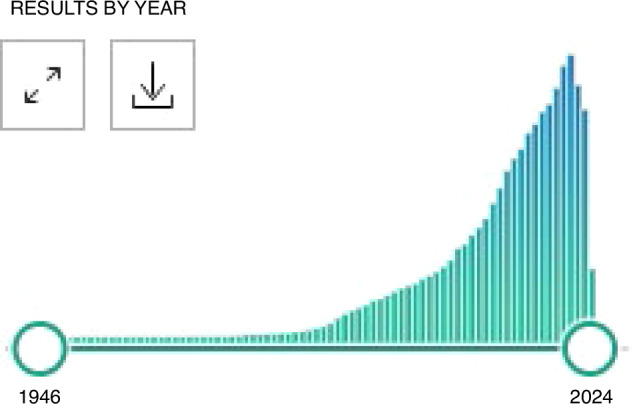
Histogram of publications including the medical subject heading (MeSH) term “biomarker,” by year, indexed in PubMed, demonstrating a peak of 84,413 manuscripts published in 2021 alone.

These early efforts paved the way for exponential growth in this field of research. The first publication in PubMed using the medical subject heading (MeSH) term “biomarker” was published in 1946. Not until 1959 did the number of published manuscripts with the MeSH term biomarker exceed 10 per year. By 1980, however, over 1000 manuscripts with this term had been published. Fueled by the attention of federal government agencies and an expanding understanding of the vital role of biomarkers in environmental health research, that number has been rapidly increasing to the present, where 84,413 manuscripts containing the MeSH term biomarker were published in 2021 alone (Fig. 3).

## Biomarkers, EndpointS, and other tools (BEST) resources

As a result of this explosion in biomarker-related publications, the Food and Drug Administration (FDA) and National Institutes of Health (NIH) Joint Leadership Council recognized a need to develop common terms for use in translational science. Their guidance came in the form of a set of common definitions that are periodically updated within the BEST (Biomarkers, EndpointS, and other Tools) Resource.^1^ The first phase of the BEST Resources project included a glossary of important definitions and clarity on the distinctions between clinical assessments and biomarkers. They provided the overall definition of a biomarker as described in the introduction section above and also delineated 7 types of biomarkers based on their use, which are listed in Table 1 along with their definitions.^1^

**Table 1:** Types of biomarkers and their definition.

**Diagnostic Biomarker** **(Biomarker of Effect)**	A biomarker used to detect or confirm presence of a disease or condition of interest or to identify individuals with a subtype of the disease.
**Monitoring Biomarker** **(Biomarker of Effect and/or Exposure)**	A biomarker measured repeatedly for assessing status of a disease or medical condition or for evidence of exposure to (or effect of) a medical product or an environmental agent
**Response Biomarker** **(Biomarker of exposure and/or effect)**	A biomarker used to show that a biological response, potentially beneficial or harmful, has occurred in an individual who has been exposed to a medical product or an environmental agent. There can be two types: Pharmacodynamic biomarker or surrogate endpoint biomarker
**Predictive Biomarker** **(Biomarker of Susceptibility)**	A biomarker used to identify individuals who are more likely than similar individuals without the biomarker to experience a favorable or unfavorable effect from exposure to a medical product or an environmental agent.
**Prognostic Biomarker** **(Biomarker of Susceptibility)**	A biomarker used to identify likelihood of a clinical event, disease recurrence or progression in patients who have the disease or medical condition of interest.
**Safety Biomarker** **(Biomarker of Susceptibility)**	A biomarker measured before or after an exposure to a medical product or an environmental agent to indicate the likelihood, presence, or extent of toxicity as an adverse effect.
**Susceptibility/Risk Biomarker** **(Biomarker of Susceptibility)**	A biomarker that indicates the potential for developing a disease or medical condition in an individual who does not currently have clinically apparent disease of the medical condition.

To allow for standard interpretation and translation across studies and clear contextual connection between the biomarker and its intended use, the resource lays out the key components of a biomarker description. To reach the stated goals, a biomarker description must contain the biomarker identity (e.g. name, anatomic location, and/or physiological characteristic), its biologic plausibility and the measurement method. The biomarker identity includes a name, a unique identifier, an acronym, a source and a type (Table 2). In the case of a molecular biomarker, the name should include both the unique identifier and acronym to ensure accurate cross-referencing across different resources. The biologic plausibility consists of a brief description of how the biomarker is associated with the disease or condition of interest and gives context to its intended use. Lastly, the measurement method to quantify the biomarker is necessary to compare biomarkers between populations and studies. All of this information is critical to be able to compare determinations of the biomarker between different patient populations.

**Table 2:** An example of an environmental biomarker identity would be.

**Biomarker Name**	Blood lead
**Acronym**	Pb
**Unique Identifier**	Pb
**Source**	Blood
**Type**	Molecular
**Biologic Plausibility**	Has been strongly correlated with cognitive outcome
**Measurement Method**	inductively coupled plasma mass spectrometry (ICP-MS)
**Units of Measurement**	μg/dL

## Developing a biomarker

**Fig. 4:**
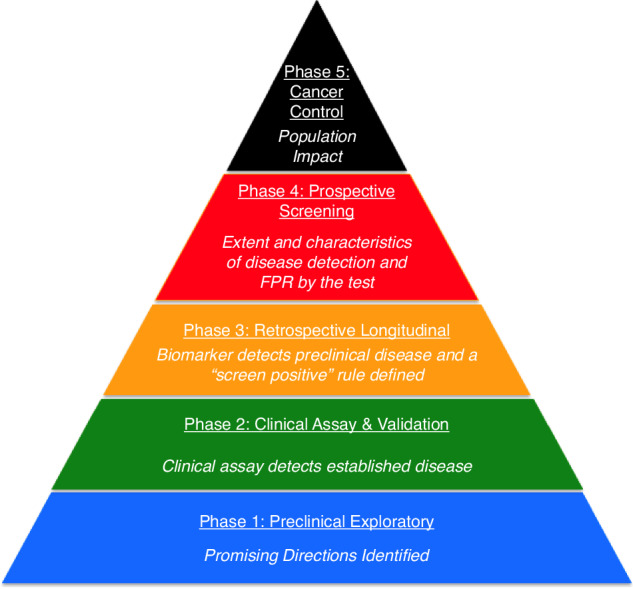
Five Steps of Biomarker Development, taken from the Early Detection Research Network, National Cancer Institute. This systematic approach to discovery, development and validation is used to help identify “winners” or “losers” among biomarkers.

Limitations of the initial BEST Resource include the lack of comprehensive data cataloging to support the use of the biomarker. Perhaps more importantly, the current version does not require the biomarker description to meet any qualitative requirements—such as an area under a receiver operating characteristic (ROC) curve—greater than a value set by the FDA-NIH Joint Leadership Council. The ROC curve signifies how well the biomarker performs, providing the probability that a biomarker would lead to correct classification of a pair of subjects: one drawn at random from all those with the outcome of interest and the other drawn at random from those without the outcome.^9^ Additionally, the next revised version (Phase 2) of the BEST Resource may describe the critical steps in developing a biomarker. Figure 4 illustrates the pathway for developing a biomarker used specifically for early cancer detection.^10^ Describing the identity of a biomarker is important, but once the biomarker has been described, it is then vital to ensure that the biomarker provides meaningful information. Importantly, these validation steps must be completed for each separate purpose of the biomarker. In short, the biochemical or imaging assay must be shown to be robust and reliable in preclinical and exploratory studies. While preclinical and exploratory clinical studies may indicate that a biomarker has promise for a given use, that promise needs to be validated with clinical studies designed to assess performance characteristics in diverse patient sub-populations. A clear statement of the purpose of the biomarker must be stated, including the risks and benefits, which may differ in biomarkers for prognosis (i.e. information regarding which outcomes are likely) versus prediction (i.e. information about potential treatment benefit).

With this history and the standards of biomarker development in mind, this review will now focus on two areas: matrices with which to measure fetal/neonatal biomarkers of environmental exposure/effect, and the specific use of breath as a matrix in pediatrics.

## Fetal and neonatal biomarkers of exposure/effect

There are many different matrices (e.g. blood, urine, etc.) in which biomarkers can be measured and each contains unique inherent risks and benefits. Specifically, matrices for the measurement of biomarkers of prenatal exposure/effect fall mainly into two categories – matrices acquired during fetal life concurrently with the exposure or those available at birth.

Concurrent matrices are obtained during fetal development and can be further subdivided into invasively or non-invasively acquired matrices. As invasive biomarkers – such as those obtained from blood—have been reviewed before,^11^ we will discuss only the non-invasive biomarkers. Most non-invasive biomarkers use matrices only available after delivery, including placenta, cord blood, umbilical cord, hair, urine, saliva, vernix, and meconium.^11^ However, some non-invasive biomarkers can be analyzed concurrently with the maternal/fetal exposure using matrices available during pregnancy, such as maternal blood containing cell free DNA.^12^ Cell free DNA originating from the fetus has been found to be present as early as 10 weeks of pregnancy and remains in the blood until delivery. It was first used to detect chromosomal anomalies such as trisomy 21, 18 and 13 and identification of the sex chromosomes. It has now expanded to several other single gene disorders such as achondroplasia and Huntington’s disease but its main application continues to be primarily in prenatal genetic diagnosis. Given that cell free DNA can detect the presence of *in utero* infections^13,14^ and changes in DNA methylation are being explored as a biomarker of several diseases such as breast cancer,^15^ it is likely that cell free DNA could also be explored as a potential biomarker for in utero exposures. While many of the non-invasive biomarker matrices show promise, one novel non-invasive matrix that has not received much attention is exhaled breath.

## The promise of breath as a biomarker for environmental exposures

Exhaled breath can be used to measure several volatile organic compounds (VOCs),^16^ including lipid degradation products, aromatic compounds, thio compounds, ammonia and amines, and halogenated compounds; changes of which have been correlated with diabetes, cirrhosis, renal disease, and other disease processes.^17^ The original studies were performed using gas chromatography-mass spectrometry (GC-MS) analyses on stored breath samples. GC-MS is a sensitive approach that can measure several individual compounds; however, the laboratory processing time required to run GC-MS assays results in a delay between sample collection and results. Real-time breath analyzers – such as breathalyzers which can estimate alcohol exposure to the pregnant woman—have been available for many years and allow for immediate diagnosis.^11^ Other recent advanced technologies—including the frequency comb analyzer^18^ and electronic nose^19^—also allow for real-time results but are not able to discriminate between individual compounds.

Much of the literature surrounding breath analysis has focused on the chemicals released during bacterial or parasitic infections such as pneumonia, tuberculosis, or malaria,^20–24^ though changes in breath VOCs have also been demonstrated in asthma, liver disease, diabetes, and ischemia.^25–27^ Adapting these methods to pregnancy have shown that exhaled VOC profiles are altered in pregnant versus non-pregnant women^28^ and, in pregnant women, maternal breath has also been associated with the development of pre-eclampsia,^29^ gestational diabetes,^30^ and chorioamnionitis.^31^ For markers of environmental exposure, real-time measurement of breath biomarkers has shown promise in non-pregnant populations,^32–34^ but there is a paucity of data during pregnancy.

Concurrent analyses can provide a real-time understanding of maternal exposure, but due to the complexity of placental transfer of chemicals and a continued paucity of understanding of the fetal dose response to VOCs, maternal exposure cannot be assumed to equate to physiologically relevant fetal effects. As such, measurements of fetal exposure in the newborn may provide for a better understanding of true fetal effects. Several studies have demonstrated utility of non-invasive matrices such as meconium, hair, and urine in detecting fetal exposure to illicit drugs and/or environmental exposures.^35–38^ Transplacental passage of VOCs has been demonstrated by detection in newborn meconium,^39^ suggesting that breath VOCs may be able to provide an assessment of *in utero* exposure.

Despite apparently robust interest in the topic, however, very few studies exist on early postnatal breath analysis to detect fetal exposures. Demonstrating this disconnect, although they found 11 published reviews on the subject, the authors of a recent review on VOCs in preterm infants demonstrated only 3 original science articles assessing breath VOCs in the population.^40^ Of these, one study sampled exhaled breath in the expiratory limb of the ventilator in five infants and analyzed the VOCs by GC-MS.^41^ The other two studies sampled air within unoccupied and occupied incubators and detected the VOCs by either GC-MS^42^ or ion mobility spectrometry.^43^ These three studies suggest that breath VOC measurement could be feasible even in small preterm infants; however, the three studies combined only included a total of 32 samples, so no clear conclusions can yet be made from the existing literature in that population.

In addition to VOCs, other studies have demonstrated changes in exhaled nitric oxide^44,45^ and glutathione^46^ concentrations in newborns as well. Currently, the studies that best demonstrate the potential value of exhaled neonatal breath to detect antenatal exposure have centered around the detection of nitric oxide. One study in 241 healthy 5-week-old infants demonstrated that increased exhaled nitric oxide in neonatal breath was strongly associated with gestational exposure to nitrogen dioxide, with the strongest association when the exposure was during the third trimester.^47^ Breath nitric oxide in infants has also been associated with other air pollutants such as ammonia, particulate matter, ozone, and carbon monoxide.^48^ Other similar studies in 1-2-month-old infants demonstrated significantly lower tidal fractional exhaled nitric oxide if they were exposed prenatally and/or postnatally to cigarette smoke as compared to non-exposed infants.^49,50^ Although the exact mechanisms by which exhaled nitric oxide is decreased have not yet been fully identified, it has been theorized that cigarette smoke may inhibit nitric oxide synthase.^51^ Of note, these associations appear to be modified by seasonal variation,^48^ humidity,^52^ neonatal gene variants,^53^ maternal characteristics such as atopy,^54,55^ as well as atopy in the children, which can lead to increased – rather than decreased – exhaled nitric oxide.^56^

## Conclusion

Biomarker discovery technologies have greatly advanced in the past decades, but many significant gaps remain, especially regarding the prospect of exhaled breath as a concurrent and/or postnatal biomarker for prenatal environmental exposures. The existing literature suggests that exhaled nitric oxide in infants may be closely associated with maternal environmental respiratory exposure to tobacco smoke or air pollution, but further research is necessary to link these changes in neonatal breath to epigenetic or other pathological changes as the infant ages. Additionally, these associations appear to have several significant modifiers, and the detection of other types of maternal environmental exposures through exhaled breath remain largely unexplored.
